# Experimental and Thermal Stress Field Numerical Simulation Study on Laser Metal Deposition of Ti-48Al-2Cr-2Nb Alloy

**DOI:** 10.3390/ma17102189

**Published:** 2024-05-07

**Authors:** Xiaolei Li, Sen Zhao, Gang Yuan, Lujun Cui, Shirui Guo, Bo Zheng, Yinghao Cui, Yongqian Chen, Yue Zhao, Chunjie Xu

**Affiliations:** 1 School of Mechanical & Electronic, Zhongyuan University of Technology, Zhengzhou 450007, China; zsen1225@163.com (S.Z.); cuilujun@126.com (L.C.); chenyq@zut.edu.cn (Y.C.); 6941@zut.edu.cn (Y.Z.); 2 Henan Key Laboratory of Underwater Intelligent Equipment, 713th Research Institute of China State Shipbuilding Corporation Limited, Zhengzhou 450015, China; 3 Zhengzhou Key Laboratory of Laser Additive Manufacturing Technology, Zhengzhou 450007, China; 4 School of Materials Science and Engineering, Xi’an University of Technology, Xi’an 710048, China

**Keywords:** TiAl alloy, laser metal deposition, numerical simulation, residual stress

## Abstract

The experimental and numerical simulation analysis of a TiAl alloy by laser metal deposition technology is presented in this paper. The research examines the macroscopic morphology, microstructure, and mechanical properties of samples as laser power varies. It also delves into how the temperature field and residual stress evolve under different laser powers. The results reveal that the microstructure of samples is mainly composed of α_2_-Ti_3_Al phase and a γ-TiAl phase and that the details of the microstructure are significantly affected by laser power. As laser power increases, coarse lamellar structure content increases, corresponding to a decrease in α_2_ phase content. The deposited layer hardness ranges from 550 HV to 600 HV, and the average deposition layer hardness decreases with increased laser power. Simulation results predict the molten pool’s size, temperature, and residual stresses. A significant increase in the molten pool size is observed when the laser power exceeds 1000 W, and the measured molten pool depths correspond closely to simulation predictions. However, significant tensile stresses are generated in the deposition layer due to high cooling rates, mainly in the x direction. Cracks are observed on the surface of the deposition layer at all laser powers.

## 1. Introduction

Titanium aluminum (TiAl) alloy is an intermetallic material that has received considerable attention due to its exceptional properties, including low density, high stiffness, excellent corrosion resistance, and ability to maintaini high strength and oxidation resistance at high temperatures [1,2]. These distinctive characteristics make TiAl alloys crucial for high-temperature structural components, such as those used in aerospace, automotive, and power applications [3,4]. In the last two decades, TiAl alloy high-temperature materials have been widely studied and favored as a preferred structural material for replacing low-pressure turbine blades in gas engines, which can reduce their structural weight by 20–30% [5,6]. However, the shape and size of TiAl alloys are limited by traditional manufacturing techniques. In recent years, the rapidly advancing additive-manufacturing (AM) technology has provided a solution for producing TiAl alloy parts [6,7].

Compared to traditional manufacturing techniques, the laser metal deposition (LMD, as a type of AM) technology can manufacture complex parts without requiring a mold and has advantages in saving production time and reducing material waste [8,9]. However, in depositing TiAl alloy, the temperature gradient is significant, and large residual stresses can form, leading to cracking during the forming process [10]. Researchers have found that focusing the powder below the matrix plane can reduce the number of cracks in the sample [11]. Additionally, researchers have reduced cracks through additional processes, such as matrix preheating, optimizing process parameters, and incorporating composite structural materials [12,13,14].

TiAl alloys with Al content ranging from 45–48 at% have been shown to possess valuable engineering applications. However, the TiAl alloy typically exhibits low ductility and fracture toughness at room temperature. Structural parts made from TiAl alloy often display a non-uniform microstructure, resulting in composition segregation and dispersed mechanical properties within the workpiece [15].

LMD technology encompasses metallurgical processes that swiftly melt and solidify metal, triggering intricate physical and chemical phenomena like heat and mass transfer, convection, diffusion, and phase transition. Real-time monitoring of temperature and residual stress changes during the LMD process poses a challenge due to current technical constraints. Research suggests that a combination of numerical simulation and experimentation can refine LMD process parameters by examining the temperature field and residual stress evolution [16]. Wang Dongsheng et al. devised a numerical model for the multi-channel lap temperature field on the surface of laser-metal-deposited TiAl alloy. Their findings indicate that optimized process parameters yield high-quality deposition layers with uniform melting and low dilution rates [17]. Yang Guang et al. utilized ANSYS programming language to simulate the thermal-mechanical coupling field of laser-melted damaged parts numerically. They observed that different scanning strategies can affect the distribution of node thermal-cycle characteristics, temperature, and residual stress fields. The results indicated that partition scanning significantly reduced matrix heat accumulation and helped to reduce its residual stress [18]. In another study, Lei et al. carried out a numerical simulation of Ti-48Al-2Cr-2Nb and demonstrated that a crack-free deposition layer could be achieved by analyzing cooling rates and microstructure under different experimental conditions [19]. Further studies by B. Carcel and colleagues have found that higher cooling rates cause increased cracking. The scanning speed was reduced to achieve a crack-free deposition layer for TiAl alloy, and substrate preheating was implemented to lower the cooling rate [20]. Liu et al. studied TiAl alloy samples obtained after laser metal deposition and heat treatment. Their findings indicated that heat treatment altered the microstructure of TiAl, leading to a notable enhancement of the mechanical properties of the alloy [21]. In a recent investigation, Silja-Katharina et al. explored the influence of various preheating temperatures and process parameters on TiAl alloys. Through simulations and experimental tests, they determined that the rapid cooling rate during the deposition process predominantly caused residual stress, rendering TiAl alloy samples susceptible to cracking during multilayer deposition [22].

This study conducted experimental and numerical simulation studies on TiAl alloys using LMD technology. A single-channel deposition experiment was conducted without preheating to replicate the original cracking behavior, maintaining a constant speed of 9 mm/s and employing laser powers of 1000 W, 1200 W, 1400 W, and 1600 W. Subsequently, the molten pool size, microstructure, and microhardness of the deposited samples were assessed. Furthermore, numerical simulation was utilized to establish the deposition process, and the cross-sectional morphology of the deposition at the same position was compared with the simulation model to analyze the temperature changes in the molten pool during the deposition process and the subsequent evolution of residual stress. In addition, the stress distribution from the numerical model can be used to understand crack formation.

## 2. Materials and Methods

### 2.1. Experimental Materials and Methods

In this study, LMD technology was used to deposit on the surface of a titanium alloy (Ti-6Al-4V) substrate. The material was Ti48Al2Cr2Nb (denoted Ti4822) alloy powder. The actual content of each element in the alloy powder was Al 32.58 wt. %, Cr 2.39 wt. %, Nb 4.85 wt. %, O 0.064 wt. %, and N 0.003 wt. %, and Ti was the remainder. Prior to the experiment, the powder underwent a drying process in a vacuum-drying oven at 150 °C for 2 h. The LMD experimental setup utilized the LMD8060 equipment manufactured by Nanjing Zhongke Raycham Laser Technology Co., Ltd. (Nanjing, China). This equipment is equipped with an LDF-4000 fiber-coupled diode laser and a four-way powder-feeding 3D-printing head. The experimental procedure operates within a sealed chamber filled with inert gas (argon) to prevent oxidation. The laser energy density is calculated using a specific method [23]:

(1) $$E=\frac{P}{VD}$$

Among these values, *E* represents the laser energy density, measured in J/mm^2^, *P* denotes the laser beam power (W), *V* stands for the scanning speed (mm/s), and *D* signifies the laser beam diameter (mm), and the laser beam diameter is 3 mm. Subsequently, a single-layer, single-channel laser deposition experiment was executed with the specified parameters: laser power varied between 1000 and 1600 W, scanning speed was maintained at nine mm/s, and powder feeding rate was set to 4.8 g/min. Four deposited layers were formed using different laser powers. After the laser metal deposition experiment, the deposited layer was detected by using a detection agent to observe crack defects on the deposited surface. SiC sandpapers from 200# to 2000# were used to grind and polish on the metallographic grinding and polishing machine. The polished samples underwent immersion in a solution blend of HF, HNO_3_, and H_2_O, with a volume ratio of 1:1:8, for about 5 s to initiate corrosion. The HAOYUAN DX-2700 BH X-ray diffractometer analyzed phase composition, and the microstructure was observed by the Hitachi TM4000 Plus (Tokyo, Japan) scanning electron microscope. Ultimately, the hardness was assessed using the MINLAI HV0.3S-5Z (Shanghai, China) microhardness tester, applying a load of 2.94 N for a duration of 10 s. Each sample underwent 10 measurements to derive the average value. A cross-sectional analysis was carried out to evaluate the deposited layers’ overall mass and their geometrical characteristics (height and depth of melting).

### 2.2. Numerical Simulation Method

#### 2.2.1. Conditional Hypothesis

The LMD process deals with a complex heat exchange between a laser beam, metal powder, and matrix. In order to accurately capture the essence of LMD process, enhance computational efficiency, and simplify the myriad factors involved in the deposition process, the following fundamental assumptions are formulated based on the thermal-elastic-plastic theory of materials [24].

During the deposition process, an external continuous heating heat source in the form of a laser is employed. Assuming a Gaussian distribution of energy for the heat source and a material absorption rate of 0.4 to the laser, only thermal convection and thermal radiation are considered [19]. Other factors such as latent heat of phase change, chemical reactions, flow into the molten pool, and material loss due to gasification are disregarded. Temperature-related mechanical properties, stress, and strain are considered temperature functions. The initial stress of the material is zero, which meets the essential strengthening criterion. The material meets the Von Mises yield criterion.

#### 2.2.2. Grid Division

**Figure 1:**
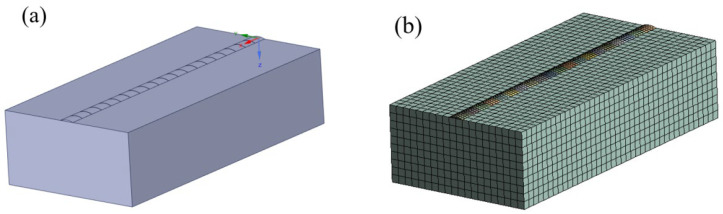
Geometric model of single-channel single-layer deposition (**a**) geometric model (**b**) grid model.

In this experiment, SolidWorks software (version 2020, USA) established a geometric model. The substrate dimensions were defined as 40 mm × 20 mm × 5 mm, while the deposition layer measured 40 mm × 3 mm × 0.4 mm, with one layer deposited, as shown in Figure 1a. The geometric model is subsequently imported into Ansys Workbench for meshing. Within the mesh model, the temperature-field part utilizes the 8-node hexahedral SOLID70 element. However, when conducting structural mechanics analysis, the SOLID70 element is transformed into the structural element SOLID45.

At the same time, to enhance the precision of the simulation outcomes, the deposition layer underwent local meshing. The total number of units after division is 8480, with 39,641 nodes, as shown in Figure 1b. Combined with the ‘life and death unit’ technology, this can effectively restore the deposition process of the sedimentary layer from scratch.

#### 2.2.3. Material Properties

Finite element simulation was conducted for laser metal deposition, employing a laser power ranging from 1000 W to 1600 W, a scanning speed of 9 mm/s, and an initial temperature of 25 °C. The substrate material used is a titanium alloy, with the deposition layer composed of TiAl alloy (Ti-48Al-2Cr-2Nb). Detailed thermal physical parameters can be found in Table 1 and Table 2.

#### 2.2.4. Heat Source Model

In the deposition process, a high-energy laser serves as an external heat source, continuously heating the material. The laser directs its intense beam onto the substrate’s surface. The heat emanates outward from the central point, with a higher density than the surrounding areas. The laser’s heat on the substrate follows a Gaussian model with a normal distribution of heat density and its formula can be expressed as [26]:

(2) $$q_{\left(r\right)}=Q\exp(-\frac{3r^{2}}{R^{2}})$$

where *q*_(*r*)_ represents the heat flux in units of J/mm^2^, *Q* represents the adequate laser power in units of W, *R* represents the radius of the laser spot in meters, and *r* is the distance between point A’ and the center of the laser spot in meters.

## 3. Results and Discussion

### 3.1. Microstructure and Melt Cell Analysis of LMD Samples

**Figure 2:**
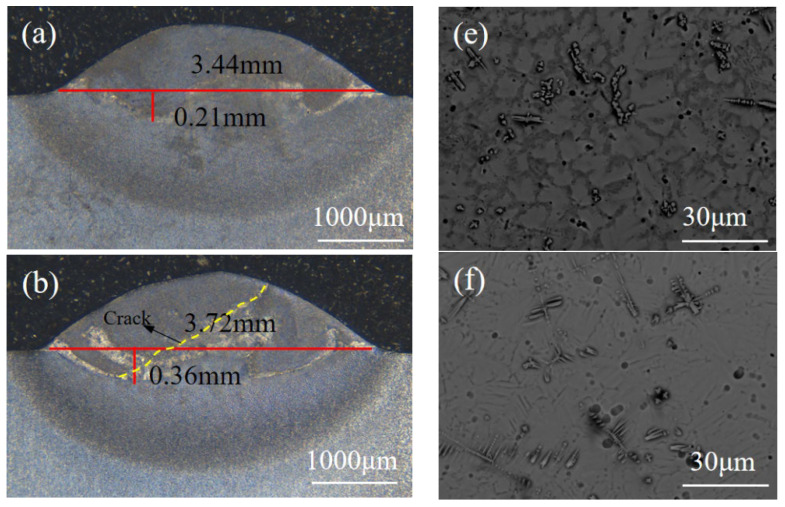

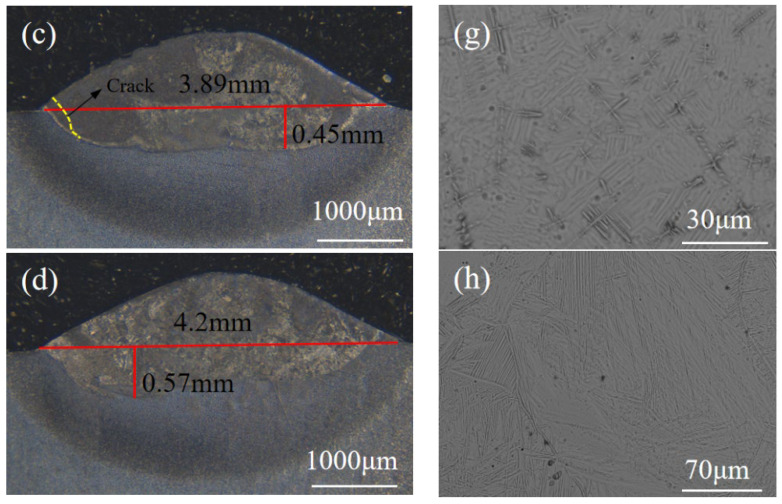
The morphology (**a**–**d**) and microstructure (**e**–**h**) of the weld pool for laser powers of 1000 W, 1200 W, 1400 W, and 1600 W, respectively.

Figure 2 illustrates the deposited specimens’ weld pool size and microstructure under various laser power conditions. The specimens’ microstructure underwent analysis and observation utilizing a scanning electron microscope (SEM), revealing that it was altered with changes in laser power. When operating at 1000 W laser power, the microstructure consists of a dendritic structure and an equiaxed crystal structure, as demonstrated in Figure 2a,e. At 1200 W and 1400 W, the microstructure is comprised of a dendritic structure and a layered structure of α_2_-Ti_3_Al phase, as illustrated in Figure 2f,g, with the layer length being relatively long at 1400 W. With a laser power of 1600 W, the microstructure exhibits a composition featuring near lamellar structures comprising α_2_-Ti_3_Al/γ-TiAl lamellae, alongside a coarse lamellar structure of the α_2_-Ti_3_Al phase depicted in Figure 2h. The lamellar structure is coarser when the laser power is 1600 W. These results reveal that the microstructure of the deposited sample displayed non-uniformity under varying laser-power conditions. SEM images indicate that the bright region comprises a coarse layered structure. In contrast, the dark region primarily consists of a combination of dendritic structure with fine equiaxed crystal and dendritic structure with fine layered structure. As the laser power escalated, the proportion of coarse layered structure in the deposited sample increased.

**Figure 3:**
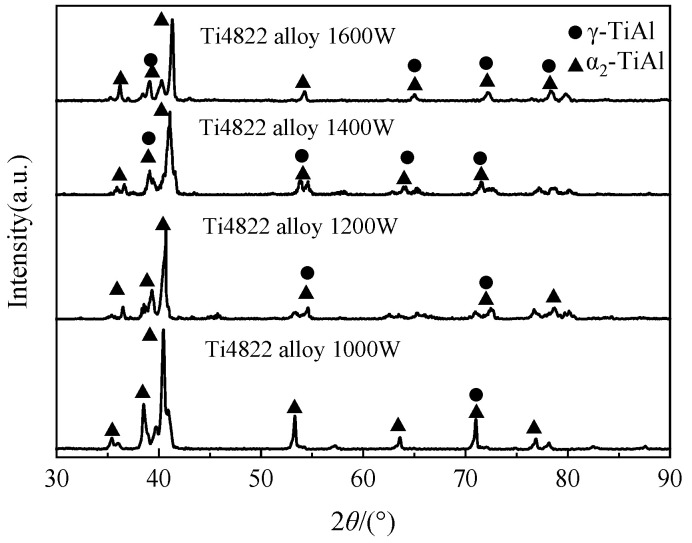
X-ray diffraction results of the deposited microstructures obtained at different laser powers.

As illustrated in Figure 3, the X-ray diffraction results indicate that the deposited samples, obtained at various laser powers, contain, primarily, a α_2_-Ti_3_Al phase and a γ-TiAl phase. The peak intensity, width, and primary peak position of the diffraction pattern of the α_2_-Ti_3_Al phase and γ-TiAl phase exhibit distinctive differences and a significant texture. Based on complete peak fitting and Rietveld refinement calculation [27], it is established that the concentration of the α_2_ phase is greater than that of the γ phase at various laser powers, with the concentration of the α_2_ phase decreasing with an increase in laser power.

It was observed that transverse cracks emerged on the surface of the deposited layer at varying laser powers. As illustrated in Figure 2b,c, these cracks extend throughout the molten pool and terminate at the fusion line. This phenomenon could be attributed to the exceptional flexibility of TC4, which effectively impeded crack propagation into the heat-affected zone [28]. When utilizing a laser power of 1200 W and a scanning speed of 9 mm/s, the maximum crack length observed in the cross-section of the molten pool is recorded at 2.1 mm. The crack length is directly proportional to the residual stress and is caused by tensile residual stress that exceeds the material’s yield strength. With an increment in laser power from 1000 W to 1600 W, the molten pool’s dimensions widen and deepen. Nonetheless, at 1000 W and 1200 W, the insufficient energy density of the laser heat source leads to inadequate metallurgical bonding between the deposited layer and the substrate. Table 3 illustrates the hardness changes within the deposited layer’s molten pool area. Compared to the traditional cast TiAl alloy, the hardness of deposited layers obtained through LMD technology significantly increases, mainly due to the alteration in microstructure. The hardness range of the LMD deposited layer was between 550 and 600 HV. An increase in laser power decreases the average hardness value of the deposition layer sample. As the laser power increases, resulting in more heat absorbed by the molten pool within a given time frame, the cooling rate slows down. This prolongs the cooling time and increases the injection and grain growth, ultimately forming coarser microstructure grains and decreasing microhardness value. During the LMD process, the phase content in the molten pool also changes. The content of the α_2_ phase decreases as the laser power increases. These results indicate that the α_2_ phase is harder.

### 3.2. Thermodynamic Analysis of the LMD Process

#### 3.2.1. Molten Pool Size

**Figure 4:**
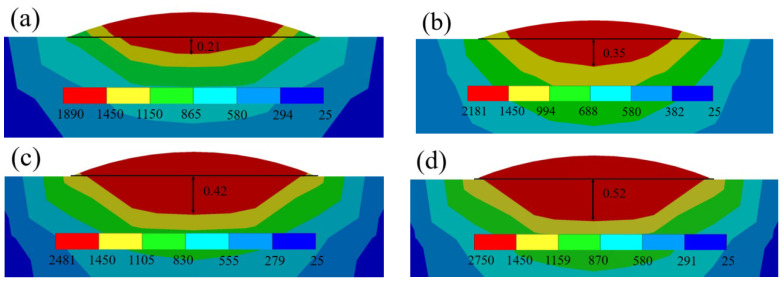
Displays of the morphology of the molten pool with laser powers of 1000 W (**a**), 1200 W (**b**), 1400 W (**c**), and 1600 W (**d**).

In order to explore how laser power affects the temperature and stress fields within the deposited layer, laser powers of 1000 W, 1200 W, 1400 W, and 1600 W were chosen, while maintaining a scanning speed of 9 mm/s. Figure 4 shows the molten pool’s morphology in the deposition layer’s cross-section. The contour’s color denotes the molten pool area, representing different temperatures. Specifically, the red color indicates the liquidus area where the temperature exceeds that of the TiAl alloy. The depth of the red area within the molten pool is quantified and compared with the morphology of deposited layers.

**Figure 5:**
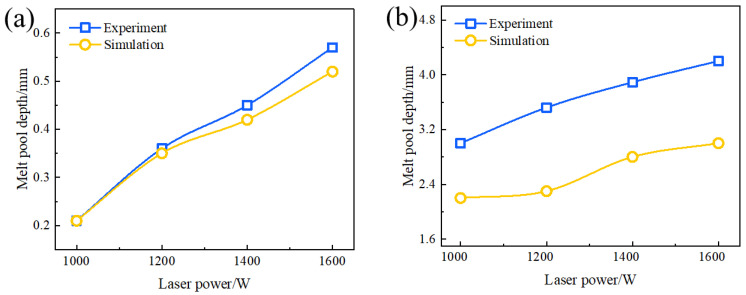
Influence of laser power on penetration (**a**) and width (**b**).

It can be seen in Figure 5 that when P = 1000 W, the measured depth of the weld pool is consistent with the simulation value. As the laser power escalates from 1000 W to 1600 W, the depth of the weld-pool temperature field shifts from 0.21 mm to 0.52 mm. Furthermore, the simulation outcomes align with the experimental data, affirming the reliability of the numerical simulation. However, the experimentally measured width of the weld pool exceeds the simulated value, a divergence likely stemming from assumptions made in the thermodynamic analysis.

#### 3.2.2. Temperature Field

**Figure 6:**
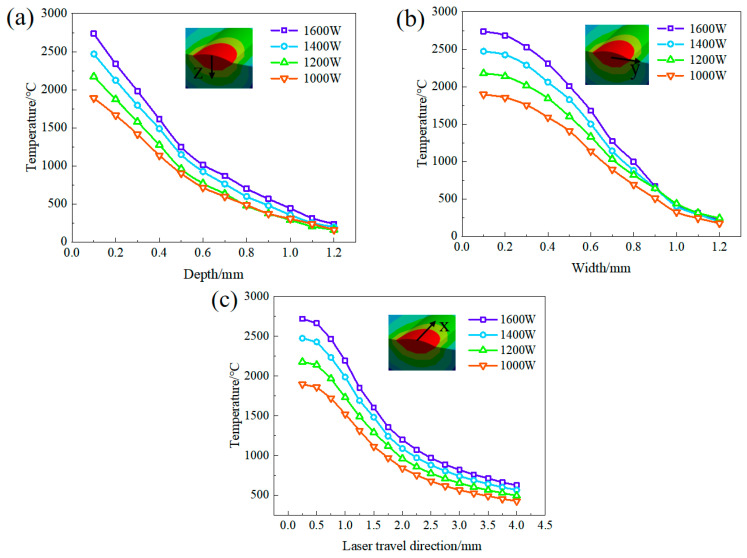
Temperature variations in the depth direction (**a**), width direction (**b**), and laser-moving direction (**c**) of the molten pool under different laser powers.

Simulation findings indicate that as the laser head progresses along the *x*-axis during the forming process, both the temperature-field distribution and the molten pool advance, resulting in the gradual enlargement of the heat-affected region. Due to the laser’s high energy density, the powder swiftly liquefies upon exposure to laser radiation, with the resulting heat diffusing along the substrate periphery. Additionally, as a result of heat transfer, the temperature near the molten pool also significantly increases. In the analysis of the temperature field, it was noted that the maximum temperature consistently resides at the center of the molten pool within the heat-source loading zone. Moreover, varying laser powers result in distinct maximum temperatures of the molten pool surface. Precisely, when subjected to power ratings of 1000 W, 1200 W, 1400 W, and 1600 W, the maximum temperatures of the molten pool surface were measured to be 1890 °C, 2181 °C, 2481 °C, and 2750 °C, respectively. It is noticeable that the temperature diminished as one moved away from the center of the laser. The melting point of the TiAl alloy is approximately 1460 °C, whereas that of the titanium alloy is 1668 °C. The temperature within the molten pool surpassed that of both the substrate and the deposition layer, creating the possibility of impressive metallurgical bonding. Figure 6 illustrates the temperature-change curves along the x, y, and z directions. Figure 6a shows the temperature distribution curve in the z-direction. Temperature data are extracted from the central surface of the molten pool to a depth of 1200 μm, encompassing both the molten pool and the heat-affected zone. The temperature decreases rapidly from the surface of the weld pool to the substrate for all laser powers. Additionally, elevating the laser power at a constant speed leads to a greater temperature gradient. The temperature distribution varies significantly along the *y*-axis compared to the other two directions.

#### 3.2.3. Cooling Rate

The cooling rate encountered during LMD was determined from the transient thermal analysis of the molten pool surface. The TiAl alloy has liquid and solidus temperatures of 1460 °C and 1440 °C, respectively. Within this restricted temperature range, it is assumed that the temperature change over time follows a linear pattern. Equation [29] describes the cooling rate at the onset of solidification.

(3) $$\frac{\partial T}{\partial t}=\left|\frac{T_{S}-T_{L}}{t_{s}-t_{l}}\right|$$

*T_L_* and *T_S_* are used to denote the liquidus and solidus temperatures determined at locations tl and ts, respectively.

The cooling rate was calculated by examining the temperature–time data of the molten pool surface. The highest cooling rate is observed on the surface, while the slowest occurs at the bottom of the pool. Table 4 shows the cooling rates of four power settings. As the power increases from 1000 W to 1600 W, the cooling rate decreases from 5.1 × 103 °C/s to 3.86 × 103 °C/s.

The deposited layer has a low temperature gradient and a high cooling rate when the laser power is set at 1000 W. The high cooling rate is directly related to the deposited layer’s microstructure, microhardness, and crack. For example, a higher cooling rate leads to a refined microstructure, affecting the deposited layer’s microhardness. At the same time, the faster cooling rate efficiently produces residual stress in the melting zone and induces cracks in the deposition layer. Based on this, predicting the cooling rate of the molten pool can better control the microstructure and cracking tendency.

#### 3.2.4. Residual Stress

**Figure 7:**
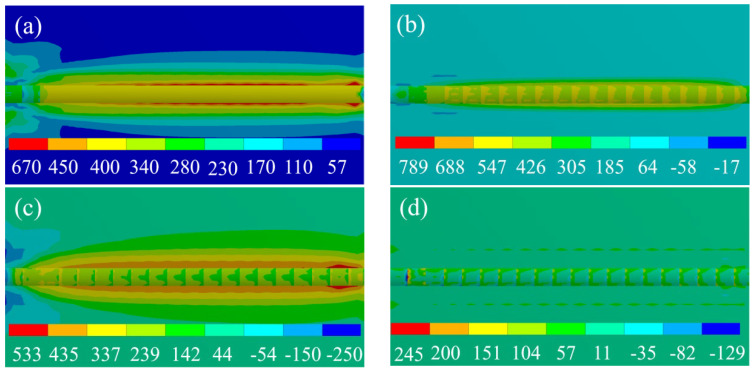
Residual stress distribution in different directions when laser power is 1400 W (**a**) principal stress (**b**) x direction stress (**c**) y direction stress (**d**) z direction stress.

To study the stress variation rules of the TiAl alloy deposition layer, numerical simulation analysis was carried out by indirect coupling based on temperature-field calculation. Since the substrate was placed horizontally on the workbench during the experiment, the degree of freedom of the substrate model was zero during the simulation process. In the LMD process, a significant cooling rate is quickly produced due to the rapid material melting and solidification, serving as the primary cause of stress. As depicted in Figure 7, the diagram of residual stress distribution at a laser power of 1400 W reveals that the primary concentration of residual stress occurs at the interface between the substrate and the deposited layer, with a maximum value reaching approximately 670 MPa. Based on the stress simulation calculations and comparisons conducted across the x, y, and z directions, it is observed that the principal residual stress lies in the x direction. The maximum residual stress approaches approximately 789 MPa, surpassing the material’s yield strength (400 MPa). Consequently, this leads to cracks forming perpendicular to the scanning direction on the surface of the deposited layer. In addition, similar stress behavior is observed in the simulation of laser power from 1000 W to 1600 W. As laser power increases from 1000 W to 1600 W, stress decreases from 720 MPa to 620 MPa, and microcracks also occur in all samples.

**Figure 8:**
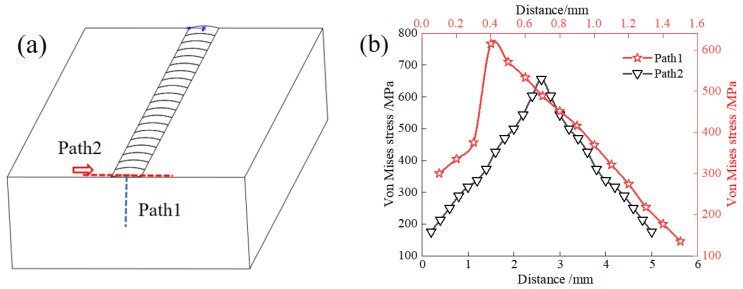
Stress distribution along different paths (**a**) and different paths (**b**) on the cross-section of the sedimentary layer.

Under the laser power of 1400 W, Vertical Path 1 and Horizontal Path 2 stress analyses were carried out, respectively, by selecting different path nodes on the cross-section of the deposition layer. The selected node path is illustrated in Figure 8a. Figure 8b displays the stress distribution curves of various paths. The residual stress of Path 1 increases first and then decreases as the depth of the deposited layer increases. At the junction of the deposited layer and the substrate, the maximum tensile stress measures approximately 670 MPa. Due to laser irradiation, both the matrix and metal powder particles experience a rapid temperature increase, resulting in condensation. However, the non-irradiated matrix has a low temperature, leading to a high radiation-resistance level, generating significant viscosity and, consequently, sizeable residual stress. The significant difference in thermal physical properties between the substrate and the deposition-layer material contributes to the emergence of cracks between them. The stress field distribution in Path 2 exhibits a symmetrical trend. As depicted in Figure 8b, the stress variation adheres to the Gaussian heat-flow distribution. Specifically, the laser irradiation areas present a bell-shaped structure, while the stress value in the remaining areas remains constant. Notably, the transverse stress surpasses the yield strength of TiAl alloy, resulting in microcracks along the transverse direction.

## 4. Conclusions

Experimental and numerical simulations of laser metal deposition of TiAl alloy have been studied in this paper. It is revealed that the microstructure of the samples varies according to the laser power employed, and the corresponding α_2_ phase content decreases. The deposited layer primarily comprises α_2_-Ti_3_Al and γ-TiAl phases, with an increase in the proportion of coarse lamellar structures observed as the laser power increases. The hardness of the LMD deposited layers, which ranges from 550 HV to 600 HV, decreases with higher laser power. The size of the molten pool increases with laser power, and the simulated depth is consistent with experimental data. The deposited layers produce high tensile stress, exceeding the yield strength of TiAl alloy, with the principal residual stress observed in the x direction. Cracks are observed on the surface of the deposited layer for all laser powers. The study provides valuable insights into the microstructure and properties of TiAl alloy deposited layers using LMD.

## Figures and Tables

**Table 1:** Thermophysical properties of Ti-48Al-2Cr-2Nb coating [25].

**Temperature °C**	25	127	327	527	727	927	1460	1534
**Density kg/m^3^**	3900	-	3879	-	3853	3840	-	3800
**Thermal conductuvity W/m K**	10.5	-	-	21.0	24.0	-	-	28.0

**Table 2:** Thermal physical parameters of Ti-48Al-2Cr-2Nb deposition layer [19].

**Temperature °C**	25	150	300	450	600	900
**Young’s modulus Gpa**	172	-	162	-	153	-
**Poisson ratio**	0.22	0.22	0.22	0.32	0.32	0.32
**Yield strength N/m^2^**	359 × 10^6^	-	358 × 10^6^	-	-	259 × 10^6^
**Tangent modulus Mpa**	377 × 10^6^	-	376 × 10^6^	-	-	-
**Thermal expansion 10^−6^∙K^−1^**	11.0	11.7	12.3	-	-	15.0

**Table 3:** Size of molten pool and microhardness of deposited layer under different laser powers.

Power (W)	Laser Energy Density (J/mm^2^)	Melt Pool Length (mm)	Melt Pool Depth (mm)	Hardness (HV_0.5_)
1000	37	3.44	0.21	600 ± 12
1200	44	3.72	0.36	589 ± 18
1400	52	3.89	0.45	565 ± 10
1600	59	4.20	0.57	550 ± 15

**Table 4:** Cooling rate under different laser powers.

Power (W)	Cooling Rate (103 °C/s)
1000	5.10
1200	4.90
1400	4.17
1600	3.86

## Data Availability

Data are contained within the article.
